# Relationships between anthropometric and body composition characteristics and age in Polish women over 60 as affected by their socioeconomic and health status and physical activity levels

**DOI:** 10.3389/fphys.2023.1198485

**Published:** 2023-06-27

**Authors:** Robert Podstawski, Aneta Omelan, Krzysztof Borysławski, Jacek Wąsik

**Affiliations:** ^1^ Department of Physiotherapy, Faculty of Physiotherapy, University of Warmia and Mazury in Olsztyn, Olsztyn, Poland; ^2^ Department of Tourism, Recreation and Ecology, Faculty of Geoengineering, University of Warmia and Mazury in Olsztyn, Olsztyn, Poland; ^3^ Institute of Health, The Angelus Silesius University of Applied Sciences, Wałbrzych, Poland; ^4^ Department of Kinesiology and Health Prevention, Jan Dlugosz University in Czestochowa, Czestochowa, Poland

**Keywords:** senior women, somatic features, age-related changes, socioeconomic status, health status, physical activity

## Abstract

**Background:** Little is known about changes in anthropometric and body composition (A&BC) characteristics during the aging process. Research indicates that body composition can be linked with socioeconomic status (SES), health status (HS), and physical activity (PA) levels.

**Aim:** The aim of this study was to evaluate age-related changes in A&BC characteristics in female seniors aged 60+ in view of their SES, HS, and PA levels.

**Methods:** The survey was conducted in November and December 2022 on a total of 661 female seniors. A questionnaire survey was conducted to obtain information about the participants’ socioeconomic status (chronic diseases, health status, marital status, membership in social organizations, financial status, place of residence, education). The respondents’ PA levels were assessed with the International Physical Activity Questionnaire (IPAQ), and their A&BC characteristics were determined in a bioelectrical impedance analysis with the InBody 270 body composition analyzer. The relationships between A&BC characteristics and age were evaluated based on the values of the Pearson correlation coefficient (*r*).

**Results:** The mean values of Percent Body Fat (PBF), Body Mass Index (BMI), and the waist-hip ratio (WHR) were relatively high (37.2%, 28.5 kg/m^2^, and 0.8, respectively) and indicative of overweight and gynoid obesity. A higher number of significant negative correlations between A&BC characteristics and age were observed in seniors with lower values of SES, HS, and PA, which points to more rapid involutional changes in this group of respondents. A segmental analysis also revealed significantly lower values of fat-free mass (FFM) and body fat mass (BFM) (both indicators were calculated in percentage and kg), in particular in the upper limbs, in women with lower SES, HS, and PA levels.

**Conclusion:** Environmental factors, including biological, physiological, environmental, psychological, behavioral, and social factors, are significantly associated with aging in women. Age-related changes in A&BC characteristics tend to proceed more rapidly in female seniors with low values of SES and HE and insufficient PA levels.

## Introduction

Aging is a natural and irreversible process that is accompanied by degenerative changes in morphological parameters and most physiological and psychological functions which accelerate with age (McKinnon et al., 2016; Ruiz-Montero and Castillo-Rodriguez, 2016; Rahmati-Ahmadabad et al., 2017). Most seniors experience a general decrease in functional fitness (Milanović et al., 2014) which are associated with the “geriatric giants”, namely, immobility, instability, depression, incontinence, and intellectual impairment. A prolonged decline in functional fitness leads to the gradual loss of independence, and the affected individuals require permanent and long-term care (Taylor-Piliae et al., 2010). Aging induces changes in basic anthropometric and body composition (A&BC) characteristics, which leads to an overall decline in health (Ryall et al., 2008; Colado et al., 2012; Silva Neto et al., 2019). Research indicates that body composition can be linked with socioeconomic variables and physical activity (PA) levels (Dos-Santos et al., 2001; McLaren, 2007; Ward et al., 2015; Staatz et al., 2019). Some health problems in old age are genetically conditioned, but many issues are associated with the social environment (such as the place of residence, membership in community organizations, marital status) and individual characteristics, including gender, ethnicity, PA, diet, and socioeconomic status (SES). The environments in which people live as children, combined with personal characteristics, exert a long-term impact on how they age (WHO, 2022). Age-related changes in A&BC characteristics also result from a combination of genetic, behavioral, and environmental factors (Lakerveld and Mackenbach, 2017; Sahibdeen et al., 2018). They may be related to hormonal status, ethnicity, disease incidence, diet, as well as the recommended level of PA (Guo et al., 1999; Macek et al., 2020b; Omelan et al., 2022).

Numerous research studies have confirmed that PA and a proper diet play a key role in all stages of life, and are particularly helpful in maintaining a healthy body weight and body composition (Indra, Sumaryanto, Setiawan, 2023; Makaraci et al., 2023). In the past, obesity was classified a health risk factor, but in recent years, it became recognized as the principal cardiovascular risk factor by the American Heart Association (Tao et al., 2019; Macek et al., 2020b). Moreover, demographic trends point to a steady rise in the prevalence of obesity among seniors. In this population group, obesity poses a significant health risk, especially for chronic diseases, and it is directly associated with a higher mortality rate (Villareal et al., 2015; Macek et al., 2020a). According to Eriksen et al. (2016), starting physical activity at any age can improve muscle mass or significantly slow down adverse health changes. Seniors who adopt an active lifestyle are at lower risk of many disabling conditions and chronic diseases—they enjoy better health, wellbeing, and greater independence (Macauley, 2001; Lampinen et al., 2006; Davis and Fox, 2007; Hilgenkamp et al., 2010; Awais et al., 2013; Podstawski and Omelan, 2015).

It should also be noted that changes in the contemporary family model as well as demographic changes have increased the number of seniors living in single-person households (singularization of old age) (Piekut, 2020). Most single-person households are headed by women. Women tend to outlive men, and this phenomenon is known as the “feminization of old age”. In 2020, women accounted for 53.2% of the 60+ population around the world (ESCAP, 2022). The gender discrepancy is also visible in the European Union (EU), in particular among the oldest members of the population (aged 85 years and more). In 2019, there were more than twice as many (2.1) very old women (85+) than very old men in the EU-27. As regards life expectancy at the age of 65 years, the greatest differences between the sexes were noted in the EU countries which have the lowest life expectancy for both genders, including Poland (Eurostat, 2020).

Polish seniors reside in various types of communities, have different educational status, and PA levels (Omelan et al., 2016; Omelan et al., 2020). In Poland and other countries (Marini et al., 2020), the elderly also differ in body composition which is influenced by gender (Omelan et al., 2022). An analysis of age-related changes in A&BC characteristics in women can provide valuable information for diagnosing health risks and implementing lifestyle changes to improve seniors’ quality of life. An awareness of the factors instrumental in inducing changes in A&BC characteristics and, above all, the very nature of these changes, is essential for assessing individual health status (HS), predicting the risk of deterioration, and selecting the optimal range of preventive measures (Tian et al., 2016; Macek et al., 2020). Age-induced changes in A&BC characteristics increase both the risk of cardiometabolic disorders and disability (Ding et al., 2007). The ratio of fat mass (FM) to fat-free mass (FFM) has been indicated as an essential risk factor for assessing major public health issues (Yusuf et al., 2004).

It should be emphasized that research on the health status and health-promoting behaviors (including physical activity) of the elderly has social and practical implications. The obtained data and the formulated conclusions should be used in senior policy planning. Thus, these findings can assist various institutions (state and non-state organizations involved in supporting seniors) in the process of improving the quality of life in this social group. Therefore, the aim of this study was to analyze age-related changes in A&BC characteristics of women over 60 who were divided into categories based on grouping variables relating to socioeconomic factors and individual traits.

## Materials and methods

### Participants and eligibility criteria

**FIGURE 1:**
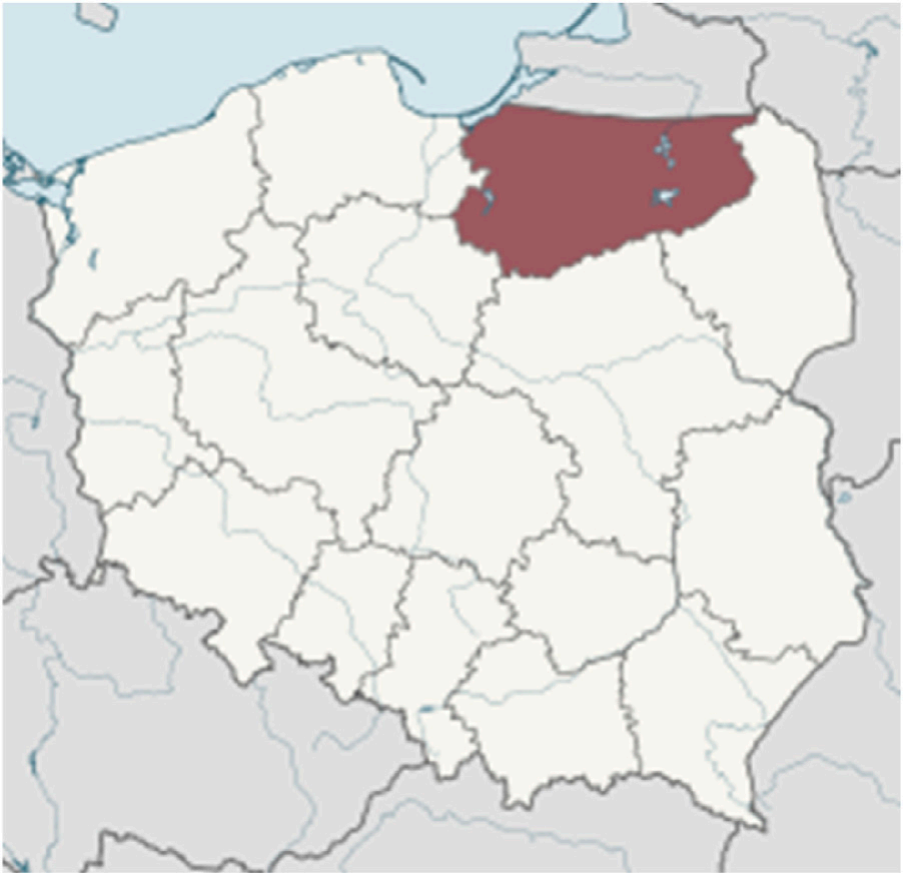
Voivodeship of Warmia and Mazury where the research was conducted.

A total of 713 female seniors (above 60 years of age) residing in north-eastern Poland were invited to the study, and 661 of those agreed to participate in the research. The participants were selected by purposive-probability sampling. The Federation of Social Work Organizations in the voivodeship of Warmia and Mazury (Figure 1) keeps a database of seniors, and it assisted the researchers in selecting the survey sample and contacting respondents. Warmia and Mazury was selected for the study for a number of reasons. This peripheral region is located along Poland’s north-eastern border. Agriculture and tourism are the main sources of income, and unemployment is relatively high, in particular outside the tourist season. The region is covered by extensive forests and agricultural land; industrial development is low, and the local road network is sparse. As a result, Warmia and Mazury is characterized by the lowest incomes and one of the highest poverty rates in Poland (Observatory of Social Integration, 2013). Young people emigrate to other Polish regions in search of employment. Warmia and Mazury has a rapidly aging population and a low fertility rate, which leads to a population decline (The Government Population Council, 2017). Elderly residents in large cities and adjacent rural areas (satellite villages) receive support from senior organizations and institutions implementing senior policies, and they have better access to healthcare services. Older people living in urban areas often belong to formal and informal community groups, participate in various activities, and are more willing to take part in surveys, which is why information about their HS is much easier to acquire. However, Warmia and Mazury is a largely rural region, and seniors residing in small towns and villages are much more difficult to access. According to *Statistics Poland* (GUS, 2020), population aging will proceed much more rapidly in Warmia and Mazury than in other Polish regions. Therefore, an analysis of age-related changes in A&BC characteristics should be conducted in this region. The study involved only female seniors because the feminization of aging can be clearly observed in Warmia and Mazury. Elderly women living in the largest city in the region (with a population of 130,000), more than ten small towns (up to 20,000), and several dozen traditional villages situated remotely from urban centers were selected for the study.

### Ethical approval

The study had been approved by the Ethics Committee of the University of Warmia and Mazury in Olsztyn (UWM), Poland (decision No. 06/2018). The participants were senior female volunteers who signed an informed consent statement.

## Procedures, data collection, and equipment

### Information about the participants’ socioeconomic and health status

The participants were surveyed with the use of a questionnaire to elicit information about their socioeconomic status (chronic illnesses, HS, marital status, membership in community organizations, financial status, place of residence, educational background). The study involved an original survey questionnaire developed by the authors. The questionnaire was developed based on a review of the literature on socioeconomic surveys, the authors’ previous experience, and survey tools that had been used by other authors to measure SES. Due to cognitive impairment resulting from old age and/or educational deficiencies, some participants had to be surveyed with the use of face-to-face interviews to facilitate communication and ensure that the questions were correctly understood.

Self-assessed HS is a general measure of health that combines physical, social, emotional, and mental health, as well as wellbeing (Doiron et al., 2015). In the questionnaire, the participants could select one of the four responses to describe their HS: 1) good, 2) rather good, 3) average, and 4) poor. However, to ensure that each group was appropriately sized, the surveyed seniors were divided into two groups with higher (good and rather good) and lower (average and poor) values of self-assessed HS. The same approach was used to classify respondents based on the results of the self-assessment of financial status.

### Assessment of PA levels

Women’s PA levels were assessed using the International Physical Activity Questionnaire (IPAQ—Polish short version) based on the average number of minutes dedicated to PA per week. The Polish version of the IPAQ has been registered by the IPAQ International Committee (Biernat et al., 2007). The energy expenditure associated with the reported activities was expressed in metabolic equivalent of task (MET) units. The MET is the ratio of the working metabolic rate to the resting metabolic rate, where 1 MET denotes the amount of oxygen utilized per minute (3.5 mL/kg/min) (Lee et al., 2011). Based on the number of METs per minute, the participants were divided into groups representing low (L < 600 METs/min/week), moderate (M < 1,500 METs/min/week), and high (H ≥ 1,500 METs/min/week) levels of activity.

### Measurement of A&BC characteristics

Body height was measured to the nearest 1 mm with a calibrated Soehlne Electronic Height Rod 5,003 (Soehnle Professional, Backnang, Germany) according to standardized guidelines. Body composition parameters, including body mass [kg] (measured to the nearest 0.1 kg), body mass index—BMI [kg/m^2^], total body water—TBW [kg], protein and mineral content [kg], body fat mass—BFM [kg], fat-free mass—FFM [kg], skeletal muscle mass—SMM [kg], percent body fat—PBF [%], waist-hip ratio - WHR, and visceral fat level—VFL, were determined by bioelectrical impedance with the InBody 270) body composition analyzer (InBody Poland, Białystok, Poland) (Alföldi et al., 2021). Body fat mass (BFM) and fat-free mass (FFM) were calculated in both percentage [%] and kilograms [kg] for the upper limbs, trunk, and the lower limbs. The following BMI classification has been proposed for persons older than 60 years: underweight = less than 23 kg/m^2^, normal/overweight = 23.0–29.9 kg/m^2^, obese = 30.0 kg/m^2^ or greater for persons both younger and older than 80 (Villareal et al., 2011; Flegal et al., 2013; Waters et al., 2013; Winter et al., 2014; Porter Starr and Bales, 2015). Very few of the surveyed seniors had a BMI below 23 kg/m^2^, and general adult population standards were applied to obtain a successfully representative sample. Upper and lower limb measurements were presented in tables as the mean values of the measurements performed in the right and left limb.

### Statistical analysis

The results were processed statistically in the Statistica 13.5 program. The relationships between the analyzed A&BC characteristics and the respondents’ age were evaluated based on the values of the Pearson correlation coefficient (*r*), separately for each category of grouping variables, at a significance level of *p* ≤ 0.05.

## Results

The participants’ A&BC characteristics and mean age in groups based on categories of grouping variables are presented in Tables 1 and 2, respectively.

**TABLE 1:** Descriptive statistics of anthropomorphic and body composition characteristics in senior women.

Characteristics	Mean	SD	SEM	Min	Max
Overall A&BC analysis					
Age [years]	68.69	6.36	0.25	61	91
Body height cm]	161.00	5.66	0.22	142	178
Body mass [kg]	78.88	13.21	0.51	37.4	132.4
PA [METs/min/week]	777.6	682.8	26.6	0	3,564
TBW [kg]	33.60	4.33	0.17	20.5	50.7
Proteins [kg]	8.94	1.15	0.04	5.5	13.5
Minerals [kg]	3.21	0.41	0.02	2.0	4.8
BFM [kg]	28.12	9.37	0.36	2.4	67.9
FFM [kg]	45.76	5.87	0.23	28.1	68.8
SMM [kg]	24.98	3.48	0.14	14.4	38.6
BMI [kg/m^2^]	28.49	4.84	0.19	16.8	51.7
PBF [%]	37.22	6.94	0.27	4.2	54.2
WHR	0.92	0.08	0.01	0.58	1.19
VFL	13.12	4.68	0.18	1.0	30.0
**Segmental A&BC analysis**					
FFM upper limb [kg]	2.38	0.46	0.02	0.9	4.1
PFFM upper limb [%]	105.41	12.93	0.50	63.4	152.9
BFM upper limb [kg]	2.25	1.11	0.04	0.1	8.7
PBF upper limb [%]	240.57	120.59	4.27	42.7	871.2
FFM trunk [kg]	20.38	2.80	0.11	11.3	30.6
PFFM trunk [%]	100.71	7.18	0.28	76.9	126.0
BFM trunk [kg]	14.04	4.45	0.17	2.1	31.4
PBFM trunk [%]	268.29	85.91	3.34	46.8	582.8
FFM lower limb [kg]	6.85	1.06	0.04	3.6	11.9
PFFM lower limb [%]	96.65	8.49	0.33	72.2	142.9
BFM lower limb [kg]	4.18	1.45	0.06	0.5	11.9
PBFM lower limb [%]	175.89	62.09	2.41	47.1	509.2

***Notes:*** SEM, standard error of the mean; SD, standard deviation, PA—physical activity level; BFM, body fat mass; PBFM, percent body fat mass; FFM, fat-free mass; PFFM, percent fat-free mass; SMM, skeletal muscle mass; PBF, percent body fat; WHR, waist-hip ratio; VFL, visceral fat level.

**TABLE 2:** Mean age in groups based on categories of grouping variables.

Characteristics		Mean	SD	t	p
Marital status	Single	70.81	6.58	9.78	<0.001
	Married	66.28	5.14		
Membership in a community organization	No	68.27	6.69	1.16	ns
	Yes	68.89	6.21		
Financial status	Lower	69.54	6.52	4.00	<0.001
	Higher	67.57	5.98		
Place of residence	Rural	69.34	7.49	1.91	ns
	Urban	68.36	5.71		
Education	Lower	70.02	7.30	3.57	<0.001
	Higher	68.11	5.82		
Chronic illness	Yes	69.57	6.61	4.46	<0.001
	No	67.35	5.72		
Self-assessed health status	Poor or average	69.54	6.35	5.24	<0.001
	Good or very good	66.80	5.97		
PA [MET/min/week]	>600	67.86	5.84	3.31	<0.001
	≤600	69.48	6.74		
BMI [kg/m^2^]	normal	68.92	6.65	1.06	ns
	overweight	68.95	6.19		
	obesity	68.17	6.36		

The mean BMI in the studied population was 28.5 kg/m^2^, which indicates that the examined seniors were at the upper end of the overweight range according to the WHO classification. The mean WHR was above 0.8, which is indicative of gynoid (pear-shaped) obesity (Singht, 1994; Czernichow, Kengne, Stamatakis, Hamer, and Batty, 2011). The mean PBF (37.2%) was also above the reference value for the female population aged 60–79 (24%–35%). The respondents were not divided into distinct age groups based on factors such as membership in community organizations, place of residence, or BMI. In the studied population, single women, women with a lower financial status, less educated women, women with chronic illnesses, women with average or poor self-assessed HS, and women with insufficient levels of PA were significantly (*p* < 0.001) older.

Table 3 presents the correlations between the participants’ age and A&BC characteristics based on the following indicators or predictors of HS: chronic illness, HS, PA levels, and BMI categories. The higher the negative values of the correlation coefficient, the more severe the age-related involutional changes.

**TABLE 3:** The influence of chronic illness, health status, physical activity level, and BMI on the strength of the correlations (Pearson’s *r*) between age and the examined A&BC characteristics in senior women.

Characteristics		Chronic illness		Self-assessed health status		MET		BMI		
		Yes (n = 397)	No (n = 264)	P/AVR (n = 455)	G/VG. (n = 206)	≤600 (n = 333)	>600 (n = 328)	<25 (n = 154)	25–29.9 (n = 288)	≥30 (n = 219)
Overall analysis of anthropometric and body composition characteristics										
Body height cm]	*r*	**−.20**	ns	**−.21**	ns	**−.15**	**−.22**	ns	**−.17**	**−.28** **<.001**
	*p*	**<.001**		**<.001**		**.006**	**<.001**		**.004**	
Body mass [kg]	*r*	**−.17** **.001**	−.12 .061	**−.18** **<.001**	ns	**−.28** **<.001**	−.11 .056	ns	**−.12** **.039**	**−.23** **.001**
	*p*									
PA [MET/min/week]	*r* *p*	**−.22** **<.001**	ns	**−.15** **.001**	**−.15** **.027**	X	X	ns	**−.22** **<.001**	**−.40** **<.001**
TBW [kg]	*r* *p*	**−.13** **.006**	ns	**−.15** **.002**	ns	**−.18** **.001**	−.10 .069	ns	ns	**−.22**
										**.001**
Proteins [kg]	*r*	**−17**	ns	**−.18**	ns	**−.21**	**−.13**	ns	ns	**−.26** **<.001**
	*p*	**<.001**		**<.001**		**<.001**	**.015**			
Minerals [kg]	*r* *p*	**−.13** **.007**	ns	**−.15** **.002**	ns	**−.16** **.004**	**−.11** **.046**	ns	ns	**−.22**
										**.001**
BFM [kg]	*r*	**−.15** **.004**	−.11 .072	**−.16** **<.001**	ns	**−.28** **<.001**	ns	ns	**−.13** **.039**	**−.16** **.018**
	*p*									
FFM [kg]	*r* *p*	**−.15** **.004**	ns	**−.15**	ns	**−.18**	**−.11**	ns	ns	**−.23** **.001**
				**.001**		**.001**	**.050**			
SMM [kg]	*r* *p*	**−.18** **<.001**	ns	**−.18** **<.001**	ns	**−.20** **<.001**	**−.14** **.013**	ns	ns	**−.26**
										**<.001**
BMI [kg/m^2^]	*r*	**−.10**	ns	**−.11**	ns	**−.25**	ns	X	X	X
	*p*	**.048**		**.020**		**<.001**				
PBF [%]	*r*	−.09	ns	**−.12**	ns	**−.21**	ns	ns	ns	ns
	*p*	.057		**.012**		**<.001**				
WHR	*r*	**−.31**	ns	**−.33**	**−.14**	**−.31**	**−.28**	ns	**−.32**	**−.35** **<.001**
	*p*	**<.001**		**<.001**	**.047**	**<.001**	**<0.001**		**<.001**	
VFL	*r* *p*	**−.16** **.002**	ns	**−.18** **<.001**	ns	**−.26** **<.001**	−.10 .083	ns	**−.13** **.033**	**−.23** **.001**
**Segmental analysis of anthropometric and body composition characteristics**										
FFM upper limbs [kg]	*r* *p*	**−.25** **<.001**	**−.12** **.050**	**−.24** **<.001**	ns	**−.26** **<.001**	**−.20** **<.001**	ns	**−.16** **.006**	**−.36** **<.001**
PFFM upper limbs [%]	*r* *p*	**−.23** **<.001**	ns	**−.20** **<.001**	ns	**−.21** **<.001**	**−.16** **.003**	ns	**−.13** **.028**	**−.29** **<.001**
BFM upper limbs [kg]	*r* *p*	**−.14** **.006**	−.11 .072	**−.15** **.001**	ns	**−.26** **<.001**	ns	ns	**−.20** **.001**	**−.14** **.037**
PBFM upper limbs [%]	*r* *p*	**−.11** **.027**	ns	**−.12** **.011**	ns	**−.24** **<.001**	ns	ns	**−12** **.035**	ns
FFM trunk [kg]	*r*	**−.30**	**−.15**	**−.29**	**−.14**	**−.29** **<.001**	**−.25** **<.001**	ns	**−.21** **<.001**	**−.41** **<.001**
	*p*	**<.001**	**.014**	**<.001**	**.050**					
PFFM trunk [%]	*r* *p*	**−.29** **<.001**	**−.14** **.019**	**−.25** **<.001**	**−.18** **.010**	**−.25** **<.001**	**−.22** **<0.001**	ns	**−.19** **.001**	**−.34** **<.001**
BFM trunk [kg]	*r*	**−.19**	**−.13**	**−.22**	ns	**−.29**	**−.14**	ns	ns	**−.29**
	*p*	**<.001**	**.037**	**<.001**		**<.001**	**.013**			**<.001**
PBF trunk [%]	*r*	**−.15**	ns	**−.17**	ns	**−.27**	ns	ns	ns	**−.17**
	*p*	**.003**		**<.001**		**<.001**				**.010**
FFM lower limbs [kg]	*r*	**−.13**	ns	**−.13**	ns	**−.18**	ns	ns	ns	**−.15**
	*p*	**.010**		**.005**		**.001**				**.025**
PFFM lower limbs [%]	*r*	ns	ns	ns	ns	ns	ns	ns	ns	ns
	*p*									
BFM lower limbs [kg]	*r*	ns	ns	ns	ns	**−.24**	ns	ns	ns	ns
	*p*					**<.001**				
PBF lower limbs [%]	*r*	ns	ns	ns	ns	**−.21**	ns	ns	ns	ns
	*p*					**<.001**				

***Notes:*** P—poor, AVR, average, G—good, VG, very good. ns—no significant differences. Only significant (bold) and near significant (normal font) values were considered, X—no statistical analyses.

In general, a much higher number of significant correlations pointing to an age-related decrease in the analyzed characteristics was noted in groups with chronic illnesses, average or poor self-assessed HS, insufficient PA levels, overweight or obesity. In turn, in groups without chronic illnesses, with good or very good self-assessed HS, sufficient PA levels, and normal BMI, age-related involutional changes were less advanced, as demonstrated by a much smaller number of significant correlations and lower values of correlation coefficients in comparison with the groups characterized by more severe health deficits.

Similar observations were made based on the results of the segmental analysis. A clear age-related decrease in selected anthropometric characteristics was noted in groups with chronic illnesses, lower self-assessed HS, insufficient PA levels, and excessive BMI. Interestingly, these changes were more pronounced in the upper limbs and the trunk, whereas in the lower limbs, significant age-related changes were associated mainly with insufficient PA levels and were manifested by less desirable values of FFM (r = −.18, *p* < 0.001), BFM (r = −.24, *p* < 0.001), and percent BFM (r = −.21, *p* < 0.001).

The impact of socioeconomic factors such as marital status, membership in community organizations, financial status, place of residence, and education on A&BC characteristics is presented in Table 4. An age-related decline in A&BC characteristics was more frequently observed in seniors who were married, belonged to community organizations, had a lower financial status, lived in cities, and were better educated, and the values of correlation coefficients also tended to be higher in these groups. The segmental analysis also revealed a much higher number of significant age-related changes in the upper limbs. The absence of significant changes in the trunk and lower limbs was not associated (in the vast majority of cases) with marital status (except for lower limb FFM in singles: r = −.13, *p* = .018), membership in community organizations, higher financial status (except for lower limb FFM: r = −.13, *p* = .028), or living in a rural area (except for lower limb FFM: r = −.16, *p* = 018).

**TABLE 4:** The influence of socioeconomic factors on the strength of the correlations (Pearson’s *r*) between age and the examined anthropomorphic and body composition characteristics.

Characteristic		Marital status		Membership in an organization		Financial status		Place of residence		Educational attainment	
		Single (n = 350)	Married (n = 311)	No (n = 209)	Yes (n = 452)	Lower (n = 374)	Higher (n = 287)	Rural (n = 217)	Urban (n = 444)	Lower (n = 200)	Higher (n = 461)
Overall analysis											
Body height cm]	*r* *p*	**−.15** **.004**	**−.23** **<.001**	**−.19** **.005**	**−.17** **<.001**	**−.19** **<.001**	**−.13** **.023**	**−.13** **.047**	**−.22** **<.001**	−.13 .061	**−.20** **<.001**
Body mass [kg]	*r* *p*	−.10 .064	−.11 .057	ns	**−.15** **.002**	**−.15** **.005**	−.11 .076	ns	**−.19** **<.001**	−.14 .054	**−.17** **<.001**
PA [MET	*r* *p*	−.09 .078	**−.19** **.001**	−.13 .072	**−.20** **<.001**	**−.19** **<.001**	**−.12** **.045**	**−.24** **<.001**	**−.12** **.013**	ns	**−.18** **<.001**
TBW [kg]	*r* *p*	ns	**−.13** **.022**	ns	**−.12** **.014**	**−.13** **.010**	ns	ns	**−.19** **<.001**	ns	**−.16** **.001**
Proteins [kg]	*r* *p*	ns	**−.15** **.006**	−.11 .087	**−.15** **.002**	**−.17** **.001**	ns	ns	**−.22** **<.001**	ns	**−.19** **<.001**
Minerals [kg]	*r* *p*	ns	**−.15** **.009**	ns	**−.11** **.021**	**−.14** **.008**	ns	ns	**−.16** **.001**	ns	**−.14** **.003**
BFM [kg]	*r* *p*	−.10 .067	**ns**	ns	**−.14** **.004**	**−.12** **.024**	−.10 .077	ns	**−.15** **.002**	**−.15** **.034**	**−.13** **.007**
FFM [kg]	*r* *p*	ns	**−.14** **.016**	ns	**−.12** **.010**	**−.14** **.006**	ns	ns	**−.20** **<.001**	ns	**−.16** **<.001**
SMM [kg]	*r* *p*	−.09 .087	**−.16** **.006**	−.12 .085	**−.15** **.001**	**−.17** **.001**	ns	ns	**−.21** **<.001**	ns	**−.18** **<.001**
BMI [kg/m^2^]	*r* *p*	ns	ns	ns	−.09 .053	ns	ns	ns	**−.11** **.020**	ns	−.09 .057
PBF [%]	*r* *p*	ns	ns	ns	**−.10** **.035**	ns	ns	ns	ns	ns	ns
WHR	*r* *p*	**−.19** **<.001**	**−.32** **<.001**	**−.25** **<.001**	**−.27** **<.001**	**−.27** **<.001**	**−.25** **<.001**	**−.35** **<.001**	**−.20** **<.001**	**−.32** **<.001**	**−.24** **<.001**
VFL	*r* *p*	−.10 .055	ns	ns	**−.14** **.003**	**−.13** **.015**	−.10 .089	**−.14** **.047**	**−.11** **.017**	**−.20** **.005**	**−.10** **.032**
**Segmental analysis**											
FFM upper limb [kg]	*r* *p*	**−.12** **.022**	**−.22** **<.001**	**−.17** **.014**	**−.20** **<.001**	**−.22** **<.001**	**−.15** **.010**	**−.20** **.003**	**−.22** **<.001**	**−.22** **.002**	**−.21** **<.001**
PFFM upper limb [%]	*r* *p*	−.09 .079	**−.19** **.001**	**−.16** **.024**	**−.17** **<.001**	**−.20** **<.001**	**−.14** **.016**	**−.23** **.001**	**−.16** **.001**	**−.20** **.004**	**−.18** **<.001**
BFM upper limb [kg]	*r* *p*	**−.17** **.001**	**−.26** **<.001**	**−.22** **.002**	**−.24** **<.001**	**−.27** **<.001**	**−.19** **.001**	**−.26** **<.001**	**−.25** **<.001**	**−.26** **<.001**	**−.25** **<.001**
PBFM upper limb [%]	*r* *p*	**−.15** **.004**	**−.24** **<.001**	**−.22** **.001**	**−.23** **<.001**	**−.25** **<.001**	**−.20** **.001**	**−.32** **<.001**	**−.19** **<.001**	**−.29** **<.001**	**−.22** **<.001**
FFM trunk [kg]	*r* *p*	−.09 .080	ns	ns	**−.12** **.013**	**−.12** **.019**	ns	ns	**−.23** **<.001**	ns	**−.18** **<.001**
PFFM trunk [%]	*r* *p*	ns	ns	ns	ns	ns	ns	ns	**−.15** **.001**	ns	−.09 .053
BFM trunk [kg]	*r* *p*	ns	ns	ns	**−.13** **.006**	**−.11** **.034**	−.10 .089	ns	**−.14** **.003**	**−.15** **.037**	**−.12** **.013**
PBFM trunk [%]	*r* *p*	ns	ns	ns	**−.11** **.023**	ns	ns	ns	**−.11** **.026**	−.13 .063	−.08 .083
FFM lower limb [kg]	*r* *p*	**−.13** **.018**	−.11 .053	ns	**.18** **<.001**	**−.16** **.002**	**−.13** **.028**	**−.16** **.018**	**−.16** **.001**	**−.21** **.003**	**−.15** **.001**
PFFM lower limb [%]	*r* *p*	−.09 .078	ns	ns	**−.14** **.003**	**−.12** **.019**	ns	ns	**−.11** **.023**	**−.18** **.009**	**−.10** **.030**
BFM lower limb [kg]	*r* *p*	ns	ns	ns	ns	ns	ns	ns	**−.12** **.013**	ns	ns
PBFM lower limb [%]	*r* *p*	ns	ns	ns	ns	ns	ns	ns	ns	ns	ns

***Notes:*** Lower educational attainment—primary school and vocational school; Higher educational attainment—secondary school and university; Lower financial status—poor or average; Higher financial status—good or very good.

Bold values are statistically significant.

## Discussion

The aim of this study was to determine the severity and direction of age-related changes in the A&BC characteristics of Polish female seniors in view of their SES, HS, and PA levels. Negative correlations between age and A&BC traits were significantly more often identified in female respondents characterized by average or poor HS, insufficient PA, overweight and obesity, lower financial status, higher education, chronic illness, as well as in respondents who lived with a spouse and belonged to a social organization. A decrease in the values of selected A&BC characteristics, including body height, TBW, proteins, minerals, FFM, SMM, and WHR (larger hip circumference), can be regarded as detrimental to health. In turn, an age-related decrease in body mass, BFM, BMI, and PBF has positive implications for health.

### Whole-body analysis

Body height decreases with age in all races and both sexes, and it is related to aging changes in the bones, muscles, and joints. Past the age of 40, body height is typically reduced by around 1 cm (half an inch) every 10 years, and the decline in body stature accelerates past the age of 70 (Shah and Vilareal, 2017). In the present study, age-related height loss was greater in women with chronic illness, lower HS, insufficient PA levels, overweight and obesity, married women, women who did not belong to community organizations, women with a lower financial status, women living in cities, and, surprisingly, better educated women. The study confirmed a significant age-related decrease in body mass, excluding in subjects with good and very good HS, women with a BMI below 25 kg/m^2^, seniors who were not members of community organizations, and women living in rural areas. Women usually gain body mass until the age of 65, and a gradual reduction in body mass is observed in later years (Shah and Villareal, 2017). Overweight and obese seniors are at greater risk of functional decline, falling, loss of muscle power, malnutrition, gait and balance problems (Kıskaç et al., 2022). Therefore, the BMI is associated with functional capacity in the elderly, and the higher the BMI, the greater the risk of functional decline (Galanos et al., 1994). It can be postulated that at least some chronic illnesses affecting the examined seniors are directly related to excessive body mass and insufficient PA levels. In the current study, chronic illness and insufficient PA levels were significantly correlated with a larger number of the analyzed A&BC characteristics, and they appear to be significantly implicated in the severity of age-related involutional changes.

In seniors, body mass decreases partly because fat replaces lean muscle tissue and fat weighs less than muscle (Peter et al., 2014; Sperrin et al., 2016). Loss of muscle mass in the legs and stiffer joints compromise mobility. Excess body fat and changes in body shape can affect balance, and obese individuals at greater risk of falling (Walston, 2020). Diet (Zaragoza-Martí et al., 2020), exercise habits (McPhee et al., 2016) are significantly linked with lifetime changes in body mass. The present study did not set out to examine the influence of eating habits on body composition characteristics; therefore, the presence of such a correlation cannot be unambiguously confirmed. Research on older adults’ eating habits revealed that economic factors affect the quality of senior diets (Conklin et al., 2013). This study also demonstrated that sufficient PA levels can significantly delay aging processes, body mass and including a decrease in FFM and SMM, bone demineralization, and impaired lipid metabolism, and the values of VFL, WHR, BMI, and BFM were more desirable in physically active women (Table 2). The WHR is influenced not only by a reduction in waist circumference, but also by increased fat accumulation in the hips, which is commonly observed in elderly women (Folsom et al., 1993; Shah and Villareal, 2017). In the studied population, the mean value of PBF exceeded the norm and was determined at 37.2%.

According to Eurostat data (2020), less than 50% of Polish citizens aged 65–74 believe that they are in very good or good health, and this result is significantly below the EU average (68.5%). In the EU-27, senior women (aged 65 years and older) are also less likely to assess their HS as good or very good in comparison with elderly men (36.5% vs 43.1%). In the current study, the age-related decline in A&BC characteristics was more pronounced in seniors with poor HS and chronic health conditions. This observation gives serious cause for concern, not only because poor health can lead to physical impairment (Tasioudi et al., 2023). Chronic illnesses such as diabetes, stroke, cardiovascular disease, chronic pulmonary disease, and hypertension are significantly more likely to cause clinical depression and mood disorders in the elderly than in the general population (Norwood. 2007; Hackett and Anderson, 2005; Chang-Quan Huang et al., 2010; Izquierdo et al., 2021).

Huang, Ghose, and Tang (2020) demonstrated that in seniors, self-assessed HS is associated with financial security. The age-related decline in the financial status of elderly women is linked with poor health, higher costs of medical treatment and long-term care. Increased spending on healthcare depletes seniors’ budgets and decreases their ability to meet other life needs, which can also exert negative consequences on health. The risk of overweight and obesity increases with age. In 2017, more than one-fifth (21.2%) of seniors aged 65–74 years and 14.7% of the general population older than 16 years were overweight in the EU-27 (Eurostat, 2020). In the present study, the parameters indicative of fat accumulation (VFL, WHR, BMI, and BFM) generally decreased with age. This observation should be regarded as a positive trend, in particular since the prevalence of obesity has been rising steadily, including among the elderly, in the last two decades, especially in developed countries (Goulart and Rexrode, 2007). A study conducted in Malaysia also revealed that the prevalence of obesity decreased with aging (by 48.5% in 60–69-year-olds, by 20.8% in 70–79-year-olds, and by 11.8% in seniors aged ≥80 years), and was higher among females (42.9%) than males (38.3%) (Kyaw et al., 2022). A comparison of American seniors aged 64–74 with those aged 75+ revealed a similar trend. In 2007–2010, approximately 35% of Americans aged 65+ were classified as obese based on their BMI. In crude numbers, this represents over 8 million adults aged 64–74 years and almost 5 million adults aged 75 and older. The prevalence of obesity was lower in the 75+ (27.8%) than the 65–74 population (40.8%) (Fakhouri et al., 2012). Between 2000 and 2010, the prevalence of moderate to severe obesity in nursing homes increased from 14.7% to 23.9% (Felix et al., 2015). The number of overweight seniors can be expected to increase with population aging. Paradoxically, higher life expectancy does not imply that seniors will enjoy additional years of life in good health, but that many seniors will spend longer periods of time in chronically ill health (Vincent and Velkoff, 2010). The risk of extreme BMI (underweight or obesity) increases with age, which contributes to higher mortality in these population groups (Goulartand Rexrode, 2007).

### Segmental body analysis

The segmental body analysis revealed a significant decrease in FFM and BFM values, in particular among seniors with chronic illness, poor HS, insufficient PA levels, and relative obesity. The observed decline was particularly pronounced in the upper limbs and the trunk, whereas significant changes in the lower limbs were associated mainly with low PA levels and were manifested in the values of FFM, BFM, and PBFM. Lower values of FFM, BFM, and PBFM in the upper and lower limbs were more frequently noted in married women, women belonging to community organizations, seniors with a low financial status, and urban residents. In turn, education did not induce equally profound differences in the compared parameters, excluding FFM and PFFM in the trunk which were less desirable in city dwellers.

The age-related decline in FFM and BFM (expressed in both kg and %) was less severe in the lower limbs, probably because muscular atrophy can differ in functional groups (Vidt et al., 2012). Lower limbs enable locomotion, and seniors tend to elicit leg muscles more frequently than arm muscles (Sousa et al., 2011). Overall muscle mass declines with age, but it is proportionally greater in the lower than the upper limbs (Reimers et al., 1998; Hughes et al., 2001; Janssen and Ross, 2005; Ferrreira et al., 2009).

### Critical analysis of the results

Married seniors tend to enjoy better health than elderly people who live alone (Janghorbani et al., 2008; Mata et al., 2015; Dakowicz, 2017; Williams et al., 2017; Zueras et al., 2020). Married seniors also tend to be more active than their single counterparts (Pette et al., 2006). As previously demonstrated, adequate PA levels delay aging, which is why the results of this study are surprising. Married seniors were characterized by a more rapid decline in body composition parameters and a more rapid onset on involutional changes in comparison with single women. Markey et al. (2005) found that in comparison with single individuals, marriage was more likely to instill proactive health attitudes in men than in women. They also observed that women are socialized into nurturing roles and giving selfless support to others. Therefore, women who are wives and mothers are more likely to prioritize the health of their family members over their own health (Markey et al., 2005). In turn, single seniors can focus on their own health, and they have more time to pursue leisure activities, including PA, which deliver measurable health benefits (Nomaguchi and Bianchi, 2004; Meltzer et al., 2013). An analysis of the A&BC characteristics of the studied seniors confirms the above observations.

Senior organizations, in particular Universities of the Third Age, are very popular in Poland. Most of them operate in cities, and they offer various educational programs, including on healthy living, as well as sports and recreational activities for seniors (Omelan et al., 2019.; Omelan et al., 2020). However, the survey did not involve questions about the types of activities undertaken by senior women who were members of such organizations. Therefore, it remains unknown whether the respondents attended lectures and workshops on healthy living, participated in sports and recreational activities, or were active members of such organizations. However, judging by the results, this is unlikely. It should be noted that the surveyed seniors belong to a generation of Poles with very low levels of PA. Based on the results reported by Śniadek and Zajadacz (2010), it can be safely assumed that none of the examined elderly women had ever undertaken any form of PA on a regular basis. The present study also revealed that seniors’ financial status considerably affects their PA levels. Polish older adults with low incomes tend to have much lower PA levels (Kalbarczyk and Mackiewicz-Łyziak, 2019). Due to the lack of life-long exercise habits or poor health, the respondents could have opted for other types of activities (such as art or foreign languages) that do not exert a major influence on physical health, which could explain the rapid decline in BC parameters in women who belonged to such organizations.

As previously mentioned, most respondents who were members of senior organizations lived in cities, and urban residence was yet another factor that was associated with an age-related decline in A&BC characteristics. The respondents’ living environment, as well as factors that are responsible for differences in the lifestyles of urban and rural dwellers should be examined to explain the observed decline in BC parameters and the rate of involutional changes.

Local environmental factors can affect the quality of seniors’ lives. These include problems with pollution and access to healthy food, which are probably greater in urbanized areas. The health implications of environmental pollution have been widely researched, and pollution can be partly responsible for the more rapid advance of involutional changes in urban residents (Simoni et al., 2015; Andrade et al., 2023). Nutrition also plays an important role in this context. The survey questionnaire did not contain diet-related questions, but based on the literature (Norman et al., 2021) and the observations conducted during face-to-face interviews, it can be assumed that seniors residing in rural areas have better access to healthy, fresh, and unprocessed food. In rural Poland, most people grow their own fruit and vegetables and keep livestock after they retire, partly for financial reasons (Hornowski et al., 2020). These activities are not only a source of healthy food, but they are also a form of regular outdoor exercise with health benefits. Seniors living in cities do not have such opportunities, which could explain the more rapid progression of involutional changes among urban than rural dwellers.

Age-related changes also proceeded more rapidly in women with higher educational attainment. There is considerable evidence to indicate that better educated adults live longer and healthier lives than their less educated peers (Kemp and Karas Montez, 2020; Raghupathi and Raghupathi, 2020). In better educated seniors, this is significantly linked with higher income (Ross and Wu. 2019). Education appears to confer a lifelong advantage for healthy aging (Meeks and Murrell. 2001). Education has been shown to reduce mortality in elderly women, but it was not significantly associated with the transition from health to disability (Martin et al., 2014; Rodriguez-Laso et al., 2014). In turn, Chen and Hu (2018) found that non-disabled Chinese seniors who had attended primary school were characterized by both lower mortality and disability in comparison with older adults without any education, whereas those who attended high school and above had only a lower mortality rate. Therefore, we agree with Cutler and Lleras-Muney (2008) that the association between education and health is complicated and involves numerous factors that include (but are not limited to) the relationship between demographics and the family background. In the present study, age-related changes proceeded most rapidly in better educated seniors who lived mostly in cities, and the influence of urban residence on the decline in BC parameters was discussed above. In addition, most university graduates work office jobs that do not require physical effort. When combined with the lack of regular PA in adulthood, better education can contribute to a more rapid progression of age-related changes in comparison with less educated individuals who are more likely to work in manual occupations.

### Strengths and limitations

The strength of this study was that the authors were able to reach elderly women who have been living and working in traditional agricultural villages all their lives. Traditional rural environments are difficult to study due to logistic as well as mental constraints. Many villages in Warmia and Mazury are situated remotely from cities, and they are difficult to access. Elderly women living in rural communities are poorly educated, have little knowledge about the contemporary world, are mistrustful of scientists and reluctant to participate in research studies. Some rural respondents suffered from mental impairments and had problems with understanding all questions, which posed an additional challenge. Therefore, some seniors had to be surveyed during face-to-face interviews to elicit data for the study. The IPAQ was a certain limitation because it assesses PA levels based on the last 7 days of recalled physical activity. Physical activity levels based on IPAQ were measured using a past week questionnaire, which has important implications for the interpretation of the results. Tools that assess past-week, self-reported levels of PA are subject to seasonal fluctuations, in particular in countries where temperature, precipitation, and number of daylight hours differ across the seasons (Matthews et al., 2001). In the present study, data were collected in fall and winter months, which could explain the low PA levels of the surveyed seniors. In addition, widows were classified as single women despite the fact that these social groups have different lifestyles. Widows are characterized by significantly lower PA levels and tend to lead more sedentary lives than married women (Park et al., 2023).

## Conclusion

The environmental factors associated with SES (marital status, financial status, place of residence, education), HS (chronic illness, health condition, overweight, and obesity), and PA level significantly affect the rate of involutional changes in elderly women. The results of this study should be interpreted in a broader social context because age-related processes are significantly influenced by a group of co-existing factors (biological, physiological, environmental, psychological, behavioral, and social) that occur in various combinations and are associated with involutional changes to a varied degree. Due to multiple interactions between environmental factors, aging is a dynamic and variable process (Chmielewski, 2020). Medical advances have contributed to longevity, but under unsupportive environmental conditions, seniors may be forced to live these extra years in ill health and disability. The above applies particularly to women because their health deteriorates with age, as demonstrated in this study. Women begin to experience symptoms of physical and biological disability at around 70 years of age. They have a longer life expectancy than men, but they will be affected by numerous health conditions and will require assistance with routine daily activities in advanced age. Many elderly women are widows who live alone without any external help. For this reason, female seniors will be the main beneficiaries of care services and institutional help.

The results of this study indicate that systemic public health interventions are needed to slow down aging processes in elderly women. Such measures should include programs promoting regular PA in seniors. Neary half of the surveyed women did not perform any type of regular exercise. The current study revealed that regular PA is effective in minimizing the risk of critical illness: in women with sufficient PA levels, the age-related decrease in A&BC characteristics was less severe and observed in fewer cases than in sedentary seniors.

## Data Availability

The raw data supporting the conclusion of this article will be made available by the authors, without undue reservation.
